# Hyperkalemia-induced Leg Paresis in Primary Adrenal Insufficiency

**DOI:** 10.5811/cpcem.2017.7.35165

**Published:** 2017-10-06

**Authors:** Gregory Mansella, Frank P. Stephan, Roland Bingisser, Christian H. Nickel

**Affiliations:** * University Hospital Basel, Department of Emergency Medicine, Basel, Switzerland; † University Hospital Basel, Department of Cardiology, Basel, Switzerland

## CASE PRESENTATION

**Image 1:**
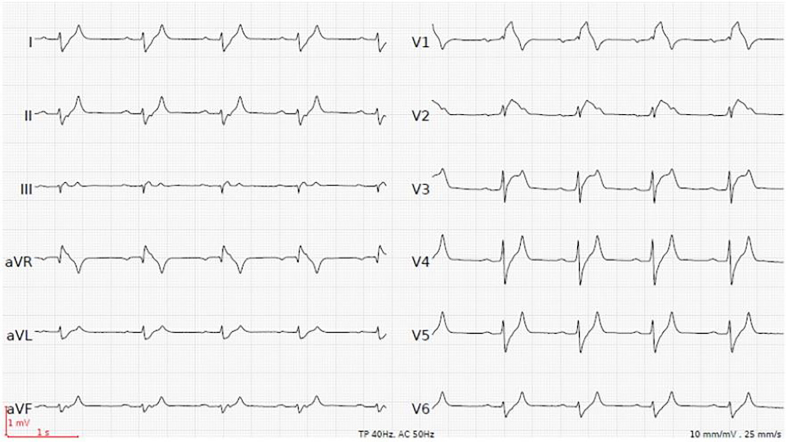
Initial electrocardiogram with a heart rate of 54 beats per minute, PR-interval 270 milliseconds, QRS-interval 122 milliseconds. Potassium level of 8.6 mmol/l.

A 49-year-old man presented to our emergency department complaining of progressive muscle weakness in his legs for three days. He had no past history of significant health issues, and denied any illicit or recreational drug use. At presentation, vital signs were normal. Physical exam revealed reduced strength (3/5) in his lower extremities but no focal deficits. Laboratory studies showed severe hyperkalemia of 8.6 mmol/l (3.6–4.8 mmol/l), hyponatremia of 130 mmol/l (135–145 mmol/l) and mild hyperchloremic metabolic acidosis. Kidney function was moderately impaired (serum creatinine 120 μmol/l (50–100 μmol/l)). The electrocardiogram (ECG) demonstrated a sinus rhythm with normal heart rate, prolonged PR- and QRS-intervals, tall peaked T-waves and type I Brugada-like pattern in leads V1 and V2 (Image 1).

**Image 2:**
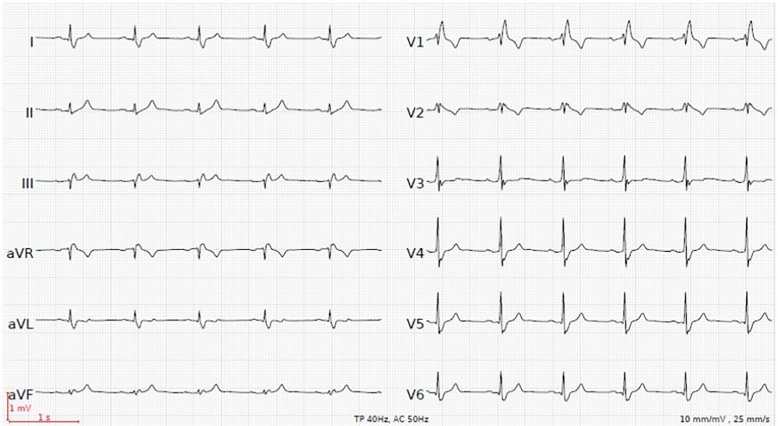
Post-treatment electrocardiogram with a heart rate of 66 beats per minute, PR-interval 170 milliseconds, QRS-interval 120 milliseconds. Return to pre-existing right bundle branch block pattern. Potassium level of 6.6 mmol/l.

Four hours after treatment with calcium gluconate, insulin with glucose, and nebulized beta-2 agonist, the ECG returned to baseline with preexisting right bundle branch block at a potassium level of 6.6 mmol/l (Image 2). Hyponatremia persisted with 130 mmol/l. The patient’s symptoms completely resolved.

Autoimmune primary adrenal insufficiency (Addison’s disease) was found as the underlying cause of this severe hyperkalemia.

## DIAGNOSIS

Hyperkalemia is found in up to 40% of patients with primary adrenal insufficiency due to mineralocorticoid deficiency.^1^ Severe hyperkalemia typically causes muscle weakness and rhythm disturbances. Muscle weakness typically begins in the legs with progression to the trunk and arms. Sphincter tone and cranial nerve function are typically not affected. Cardiac manifestations include electrocardiographic changes, conduction abnormalities and cardiac arrhythmias (Table 1).^2^,^3^ Of note, the progression and severity of ECG changes do not correlate well with the serum potassium level.^4^

This case highlights that hyperkalemia-induced muscle weakness with associated electrocardiographic changes can be the major presenting symptom in primary adrenal insufficiency. The treatment consists of lowering potassium levels and replacement of hydrocortisone and fludrocortisone.^1^

**CPC-EM Capsule**

What do we already know about this clinical entity?

Patients with primary adrenal insufficiency can present with nonspecific symptoms such as weakness. Hyperkalemia is found in up to 40% of patients with primary adrenal insufficiency.

What is the major impact of the image(s)?

The images show reversible hyperkalemia-induced electrocardiographic manifestations (including type I Brugada-like pattern) as a main finding in primary adrenal insufficiency.

How might this improve emergency medicine practice?

This case highlights that hyperkalemia-induced muscle weakness with associated electrocardiographic changes may be the only presenting symptom in primary adrenal insufficiency.

## Figures and Tables

**Table 1:** Cardiac manifestations in hyperkalemia.

Electrocardiographic changes	Conduction abnormalities	Cardiac arrhythmias
tall peaked T-waves with shortened QT-interval prolonged PR- and QRS-intervals with small or disappearing P-waves further prolongation of the QRS-interval to a sine wave pattern	right or left bundle branch block bifascicular block advanced atrioventricular block type I Brugada pattern (characterized by high takeoff ≥2 mm coved ST-segment elevation followed by a negative T-wave in at least two precordial leads)	sinus bradycardia, sinus arrest slow idioventricular rhythms ventricular tachycardia, ventricular fibrillation asystole
